# Metabarcoding and metagenomic data across aquatic environmental gradients along the coasts of France and Chile

**DOI:** 10.1038/s41597-026-06572-1

**Published:** 2026-01-13

**Authors:** Mara D. Maeke, Christiane Hassenrück, Polette Aguilar-Muñoz, Camila Aravena, Christian Burmeister, Olivier Crispi, Papa Oumar Djibril Diallo, Camila Fernández, Maëlann Gouriou, Alizée Jamont, Emile Laymand, Barbara Marie, Verónica Molina, Eva Ortega-Retuerta, Sophie Rabouille, Mazharul Islam Sajeeb, Maria Sierks, Martha Stevens, Robin Turon, Valentina Valdés-Castro, Sara Beier

**Affiliations:** 1 Biological Oceanography, Leibniz Institute for Baltic Sea Research Warnemünde (IOW), Rostock, Germany; 2 HUB AMBIENTAL UPLA, Universidad de Playa Ancha, Valparaíso, Chile; 3 Departamento de Ciencias y Geografía, Facultad de Ciencias Naturales y Exactas, Universidad de Playa Ancha, Valparaíso, Chile; 4 Centro de Investigación Oceanográfica COPAS Coastal, Departamento de Oceanografía, Universidad de Concepción, Concepción, Chile; 5 Sorbonne Université, CNRS, Laboratoire d’Océanographie Microbienne, LOMIC, Banyuls-sur-Mer, France; 6 Marine Biology Research group, Department of Biology, Ghent University, Ghent, Belgium; 7 Insitut des Sciences de la Vie, Microbiologie, University of Strasbourg, Strasbourg, France; 8 Univ Lyon, Université Claude Bernard Lyon 1, CNRS, ENTPE, UMR5023 LEHNA, Villeurbanne, France; 9 Institut de Systématique, Evolution, Biodiversité (ISYEB), Muséum National d’Histoire Naturelle, CNRS, Sorbonne Université, EPHE, Université des Antilles, Paris, France; 10 Faculty of Sciences, Ghent University, Ghent, Belgium; 11 Institute of Biological Sciences, Faculty of Mathematics and Natural Sciences (MNF), University of Rostock, Rostock, Germany; 12 Institut de Ciències del Mar, Consejo Superior de Investigaciones Científicas (ICM-CSIC), Barcelona, Spain; 13 Flanders Marine Institute (VLIZ), Oostende, Belgium; 14 CNRS, Laboratoire Microorganismes: Génome Et Environnement, Université Clermont Auvergne, Clermont-Ferrand, France

**Keywords:** Biodiversity, Water microbiology, Marine biology

## Abstract

Coastal marine environments, such as lagoons, fjords or estuaries, experience pronounced environmental variability, with fluctuations in salinity, temperature and nutrient levels shaping microbial community structure and function. These gradients result in diverse habitats, which may harbour taxonomic and genetic novelty with biogeochemical and biotechnological relevance. To explore microbial diversity and functional potential across these dynamic ecosystems, we sampled 26 sites along the coasts of France and Chile, including lagoons, estuaries, fjords, harbours, as well as coastal and offshore marine sites. Surface waters were collected from all sites, with deeper layers included at three sites. Monthly sampling at six sites in France enabled the assessment of seasonal dynamics. In total, 116 samples were processed for both metabarcoding and metagenomic sequencing yielding over 53,000 amplicon sequence variants (ASVs) and 1,372 metagenome-assembled genomes (MAGs). This dataset further includes a comprehensive gene catalogue and environmental variables such as salinity, temperature, nutrient concentrations, productivity, as well as oxygen consumption metrics collected across the different ecosystems.

## Background & Summary

Microbial communities are central to global biogeochemical cycles, and understanding their dynamics in response to environmental fluctuations is crucial for predicting global element fluxes^1,2^. Coastal marine environments provide ideal settings for studying how environmental fluctuations affect microbial community dynamics. These ecosystems are subjected to substantial physico-chemical variability due to diverse environmental and anthropogenic factors, which challenge individual taxa to adapt and thrive^3^. For instance, these environments undergo large temperature and salinity fluctuations^4^ that are driven by seasonally variable freshwater inflow or high summer evaporation rates due to their typically shallow depths, as well as environmental short-term disturbances, such as storms or heavy rainfalls. Among environmental drivers, salinity and temperature are recognized as major determinants of microbial community composition^5^.

**Fig. 1:**
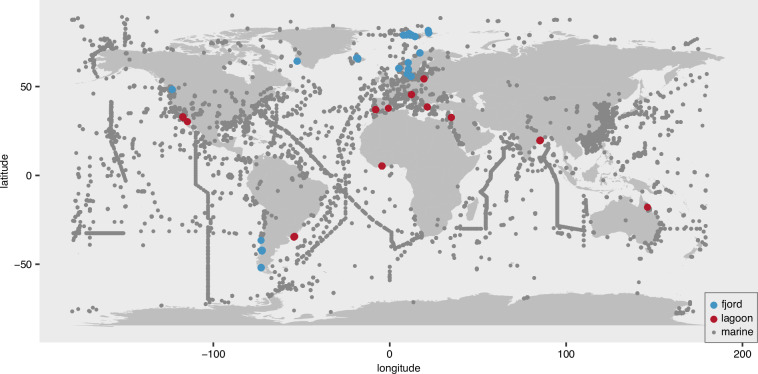
Overview of available metagenomic data from marine and/or coastal environments highlighting studies on fjords and lagoons. Grey points indicate all screened metagenomic runs (n = 39,191). Blue and red points indicate fjords (n = 398) and lagoons (n = 126). Metagenome metadata was retrieved via the ENA Advanced Search (date accessed 08.09.2025) with the following search query: ‘result = read_run & query = tax_tree(408169) AND library_source = metagenomic AND library_strategy = WGS’, downloading all returnable metadata fields in TSV format. Marine and/or coastal metagenomes were identified based on their NCBI taxon ID, while lagoon or fjord origin was determined by a string match to these keywords in any of the returned metadata fields.

Here, we present a comprehensive dataset spanning samples from coastal lagoons, salt marshes, estuaries, fjords, marine coastal sites, harbours and one offshore site. Currently, microbiome datasets from lagoons and fjords remain particularly scarce^6–11^; at present, less than 0.02% of coastal metagenomic sequencing runs deposited in the Sequence Read Archive (SRA) of the International Nucleotide Sequence Database Collaboration (INSDC) are associated with lagoons or fjords (Fig. 1). The presented dataset captures multiple environmental gradients, including terrestrial–marine transitions, salinity fluctuations, eutrophication, disturbance type and severity, as well as seasonal and geographic variation. A key objective was to include both frequently disturbed and stable environments to assess how the composition of microbial communities and their functional potential responded to environmental change across diverse coastal systems. The environmental heterogeneity within these systems creates multiple habitats that may harbour taxonomically and genetically novel microorganisms^12^. Large-scale sampling in these understudied environments increases the likelihood of uncovering both taxonomic and functional novelty, as demonstrated in other global surveys^13–15^. This novel genetic diversity holds significant promise for biotechnology, particularly in the discovery of enzymes adapted to extreme conditions^16,17^. In coastal lagoons, for example, high salinity or temperature may select for extremotolerant microorganisms whose halotolerant or thermotolerant enzymes are valuable for industrial applications, such as detergents, bioremediation, biofuel production, or food processing^18–21^.

**Fig. 2:**
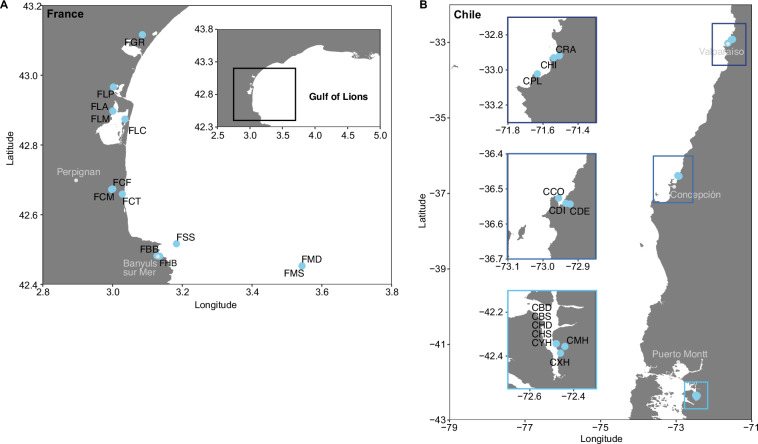
Overview maps of sampling sites along the French coast in the Gulf of Lions (**A**) and the Chilean coast (**B**). Sampling sites are shown as blue symbols; cities are shown as grey symbols with corresponding labels.

In summary, water samples were collected from 13 sites along the French coast in the Gulf of Lions (Fig. 2A) and 13 sites along the Chilean coast, spanning from Valparaiso to northern Patagonia (Fig. 2B) and covering a wide range of coastal habitats (Table S1). Six French sites (FGR, FLP, FLC, FCT, FHB, and FSS) were sampled monthly over one year to capture seasonal dynamics of microbial communities. Additional French sites (FLA, FLM, FCM, FCF, FBB, FMS, and FMD) were sampled twice to seven times throughout the sampling campaign, offering further spatial and temporal coverage along the French coast. Chilean sites were sampled once or twice during meteorological autumn. Most Chilean sites were sampled once (CBD, CBS, CCO, CDI, CHD, CHI, CHS, CPL, CRA, CXH, CYH), while sites where tidal influences were expected (CDE, CMH) were sampled twice to capture water mass variability. Except for the French offshore site FMD and two deep fjord sites in Chile (CHD, CBD) sampled at 200–500 m depth, all samples were collected from surface waters (1–5 m). First insights into French metagenomes (n = 77) focusing on fungal diversity, were provided in a recent dissertation^22^, in which fungal diversity was examined over time with respect to site variability. However, only few metagenomic sequences were assigned to Fungi across all sites.

All 116 samples were processed for both metabarcoding and metagenomic library preparation. Metabarcoding of the 16S rRNA V4-V5 gene region yielded data for 115 samples (one failed library) comprising 53,392 amplicon sequence variants (ASVs) with some samples sharing as few as three ASVs. Metagenomic sequencing from all 116 samples enabled the reconstruction of 1,372 medium to high-quality prokaryotic metagenome-assembled genomes (MAGs) following the standard of minimum information about a metagenome-assembled genome (MIMAG)^23^. The dataset also includes a gene catalogue of prokaryotic and eukaryotic genes exhibiting 31% of functional novelty. In addition, comprehensive environmental data were concomitantly collected at the time of sampling, including salinity, temperature, chlorophyll-*a*, phosphate, nitrate, nitrite, ammonium, dissolved organic carbon (DOC), humic-like fluorescent dissolved organic matter (fDOM), protein-like fDOM, respiration and leucine incorporation rates.

## Methods

### Sampling and sample processing

The Mediterranean MOLA and SOLA field stations^24^ were sampled at 5 m and 500 m depths (MOLA; FMS and FMD, respectively) and 3 m depths (SOLA; FSS). These samples were obtained using 12 L Niskin bottles and transferred to 20 L polycarbonate carboys by the Banyuls Observation Sea Service (BOSS) and the crew onboard the R/V “Néréis II”. Detailed environmental data for the stations MOLA (Mediterranean Ocean Observing System for the Environment, https://www.moose-network.fr/, accessed 22. August 2025) and SOLA (Service d’Observation en Milieu LIToral, https://www.somlit.fr/en/, accessed 22. August 2025) in France is available online. Sampling at Comau Fjord was carried out onboard the R/V “Noctiluca” operated by the Huinay Scientific Center^25^ (using 10 L Niskin bottles). At all remaining sites, surface water was collected using 20 L polycarbonate carboys either directly from the shore or in shallow lagoons up to ~200 m offshore. For each sample, 20 L of water was collected in a single carboy. Temperature and salinity were usually measured immediately after sampling using a Conductivity Pocket Meter Cond 330i (WTW GmbH, Weilheim, Germany) in France and an Orion Star Multiparameter (model A329, Thermo Scientific) in Chile. For hypersaline samples with salinity exceeding 50 psu, salinity was re-measured in the laboratory following dilution. The sample water was kept in an isothermal box to maintain *in situ* temperature during transport to the laboratory. Upon arrival at the laboratory, it was prefiltered through a nylon mesh (pore size 80 μm) to remove larger particles. The prefiltered water was used to sample aliquots for the quantification of nutrient concentrations and to measure bacterial production via leucine incorporation and oxygen consumption rates as detailed below. For downstream DNA extractions, between 100 and 6,600 mL of the prefiltered water was filtered onto a 0.22 μm pore size membrane filter (47 mm PVDF membrane filters, Millipore, MA, USA) using a peristaltic pump. The filters were shock-frozen in liquid nitrogen and stored at −80^◦^C until DNA extraction. Unfiltered sample water was used for chlorophyll-*a* (chl-*a*) and DOM measurements.

### DNA extraction

In total 117 DNA extracts were obtained from 116 samples (Table S1) from the 0.22 μm pore size membrane filters, including one duplicate filter. DNA was extracted with the QIAamp DNA Mini Kit (QIAGEN, Hilden, Germany) following the manufacturer’s instructions, with a bead-beating step (2 × 6.0 m s^−1^ for 45 s, FastPrep-24™ 5 G, MP Biomedicals, Irvine, USA) added after chemical cell lysis.

### Nutrient analyses

Silicate and inorganic nutrient concentrations including ammonium and for some samples (CBS01, CHS01, CMH01, CRA01, CXH01, CYH01, FCF01, FCM01, FCT01, FCT02, FCT03, FCT04, FCT05, FCT06, FGR03, FGR04, FLC01, FLC03, FLC04, FLP01, FLP02, FLP03, FLP04, FLP05, FLP06, FLP07) phosphate, nitrate, and nitrite and were determined using an automatic nutrient analyzer (QuAAtro39 Autoanalyzer, SEAL Analytical, Southampton, UK) as detailed in Grasshoff, *et al*.^26^. In the case of nitrite measurements, samples FHB01, FHB02, FHB03, FHB04, FHB05 and FHB06 were processed additionally with the QuAAtro39 Autoanalyzer. Phosphate, nitrate, and nitrite for the remaining samples (including FHB01-FHB05 for phosphate and nitrite) were determined on a continuous flow nutrient analyzer (AA3HR AutoAnalyzer, Bran-Luebbe, Australia)^27^. Due to a loss of sample material, the available values were obtained without replicate measurements.

### Chlorophyll-*a* measurements

Chlorophyll-*a* (chl-*a*) is an indicator for phytoplanktonic biomass. In order to estimate the phototrophic productivity of the sampled ecosystems, chl-*a* concentrations were determined from duplicate measurements using a fluorometric method described elsewhere^27^. In brief, 100–540 mL water was filtered through glass fiber filters (nominal pore size 0.7 μm, GF/F Whatman, Kent, UK) at a maximum pressure of 150 mmHg using a vacuum pump. The filters were shock-frozen in liquid nitrogen and transferred to storage at −80^◦^C. For analysis, the filters were mechanically crushed, and chlorophyll was extracted in 90% acetone in the dark at 4^◦^C overnight. After centrifugation for 10 min at 1,504 ×g and 4^◦^C, the supernatant was transferred to a new tube. Upon excitation with wavelengths of 420–450 nm, the fluorescence spectrum at wavelengths above 665 nm was measured before and after acidification with 0.3 N hydrochloric acid using a 10-AU fluorometer (Turner Designs, Sunnyvale, USA). Chl-*a* concentrations were then calculated as detailed previously^28,29^.

### Dissolved organic matter (DOM) measurements

All glassware and filters used for DOM analysis were pre-combusted (450 °C, > 5 h) to avoid carbon contamination. Collected water samples were filtered through a double layer of 25 mm glass fiber filters (nominal pore size 0.7 μm, GF/F Whatman, Kent, UK) into beakers for later conditioning and analysis.

10 mL of the filtered sample water was used for downstream DOC analyses. Sample water was transferred into glass ampoules, acidified with 85% H_3_PO_4_ (final pH 2), flame sealed, and stored in the dark at room temperature until analysis. Calibration curves were made using an acetanilide solution (C_8_H_9_NO; M = 135.17 g mol^−1^). DOC was analyzed in duplicates using the high temperature catalytic oxidation (HTCO) technique^30^ with a TOC analyzer (TOC-L, Shimadzu, Japan). A Deep Seawater Reference in sealed glass ampoules of 43–45 μmol C L^−1^, provided by the Hansell Laboratory (Univ. of Miami), was used to assess the accuracy of the measurements.

For fDOM analyses, an aliquot of the GF/F filtered sample water was freshly (within 1–3 h) analyzed in duplicates with a spectrofluorometer (FP-8500, Jasco, Japan) with a xenon lamp. Slit widths were 5.0 nm for the excitation and emission wavelengths. We characterized two groups of fluorophores using the following excitation/emission (ex/em) pairs^31^: 280 nm /350 nm (peak T), as a proxy for protein-like fDOM and 340 nm /440 nm (peak C) as a proxy for humic-like fDOM. Fluorescence intensities of the peaks are reported in Raman units (R.U.) obtained by dividing the fluorescence units by the Milli-Q blank peak area (Raman scatter) of an emission scan excited at 350 nm and recorded from 365 nm to 450 nm.

### Leucine incorporation rates

Experiments for ^3^H-labeled leucine incorporation into biomass (i.e. protein synthesis) were conducted to estimate bacterial production using the centrifugation method by Smith and Azam^32^. A working solution containing cold and radiolabeled ^3^H-leucine (specific activity: 125.6 Ci mmol^−1^) was prepared within 4 h prior to collection of samples and kept at 4^◦^C. A volume of 100 μL working solution was added to triplicates of 1,500 μL sample water (final leucine concentration: 40 nmol L^−1^). All samples were incubated in the dark at *in situ* temperature (±1^◦^C) for 90 min up to 4 h, depending on the expected productivity of the ecosystem. The incubations were terminated by adding 150 μL trichloroacetic acid (TCA, 50% v/v). As a negative control, two to three replicates of each sample were incubated by adding the 150 μL TCA stop solution before adding the leucine working solution. After terminating the incubations, samples were stored at −20^◦^C and further processed within a month.

For processing, the samples were thawed for 45 min at room temperature and centrifuged for 15 min at 16,100 × g. The supernatants were carefully sucked up with a vacuum pump at 180 mmHg and the pellets were subsequently washed by addition of 500 μL cold 5% TCA (4^◦^C). After centrifugation for 5 min at 16100 × g, the supernatant was again sucked up, and the washing step repeated with 500 µL of cold 70% ethanol (4^◦^C). Finally, 1 mL of the detection solution (Filter-Count, PerkinElmer, Waltham, USA) was added to the pellets. The radioactivity in the tubes was quantified using the scintillation counter Hidex and Mikrowin program (Version 4.44, Mikrotek Laborsysteme GmbH, Overath, Germany) with a detection time of 5 min per tube. The values of disintegrations per minute (DPM) were used to calculate the amount of incorporated tritiated leucine into cells, and further the rate of incorporation based on the sample volume and the duration of incubation. Automatic outlier removal was performed by a combination of the Hampel filter and detection of data points falling beyond the upper and lower quartiles ±9 × the interquartile range, respectively. Raw output files of the Mikrowin program are available in Sierks, *et al*.^33^ and the script to process these data, including outlier detection, is given on Zenodo^34^.

### Respiration rates

Respiration was assessed as oxygen consumption estimated in 5 mL glass vials equipped with an OxoDish using a SensorDish® Reader (PreSens, Regensburg, Germany). Tubes were filled with prefiltered sample water (6 replicates per sample) and closed with an air-tight lid. The plate reader was placed in an incubator at *in situ* temperature in the dark. Oxygen concentrations in the vials were measured every three minutes over several hours. The lowest salinity recorded across all samples measured simultaneously was used as the reference salinity in the SensorDish Reader and detected oxygen concentrations were corrected for salinities in other samples that exceeded the reference salinity as detailed by Debelius, *et al*.^35^. Oxygen consumption was then estimated by fitting a linear regression through the time series of oxygen concentration values after manually selecting a time frame in which the oxygen concentration decreased linearly. Regressions with r > −0.01 were removed to exclude incubations that were characterized by an oxygen increase due to oxygen contamination in the vial. Additionally, regressions with r falling beyond the upper and lower quartiles ±9 × the interquartile range were removed. This latter step resulted in the removal of replicates with ambiguous regressions that did not follow a linear trend. We used a respiration quotient of 1 to convert O_2_ consumption to CO_2_ production^36^. Raw output files of the SensorDish® Reader are available in Sierks, *et al*.^33^ and the script to process these data, including outlier detection, is given on Zenodo^34^.

### 16S rRNA gene amplicon sequencing and processing

The library preparation for 16S rRNA gene amplicon sequencing was carried out at Fasteris (Geneva, Switzerland). In brief, amplicon sequencing libraries were generated by PCR amplification of DNA extracts using universal primers targeting the V4-V5 region of the microbial 16S rRNA gene that cover both bacterial and archaeal diversity (515-Y: 5′-GTGYCAGCMGCCGCGGTAA-3′ and 926 R: 5′-CCGYCAATTYMTTTRAGTTT-3′)^37^. PCR products were then verified, purified, quantified, and pooled following the Fasteris in-house protocol. The pooled library was sequenced on the Illumina MiSeq V2 Nano platform (2 × 250 bp, Illumina), and demultiplexed directional paired-end reads were provided for further analyses.

Reads were primer clipped using cutadapt v4.9^38^. Further processing was performed using the package dada2 v1.30.0^39^ in R v4.3.3^40^. Forward (R1) and reverse reads (R2) were cut at 195 bp and filtered with a maximum expected error rate of 2 for both forward and reverse reads. Error rates were learned using the default error function. Reads from all samples were pooled and subsequently denoised together. Denoised reads were merged into amplicon sequence variants (ASVs), and chimeras and singletons were removed. Organelles (mitochondria and chloroplasts) were removed from the dataset using the classifications derived from the assignTaxonomy function of dada2 with the SILVA v138.1 database^41^. A more resolved taxonomic classification was then performed with the Genome Taxonomy Database (GTDB) R226 reference database^42^ with a bootstrap cut-off of 70. Rarefaction curves computed with iNEXT v3.0.1^43^ for species richness and the inverse Simpson index were used to validate whether prokaryotic diversity was sufficiently covered at available sequencing depths. All scripts and computer code developed for the analysis of the metabarcoding data is given in Maeke, *et al*.^34^.

### Metagenomic sequence processing

For metagenomic sequencing, 30 µL of DNA extract containing ~200 ng of DNA was used for library preparation at Fasteris (Geneva, Switzerland), with DNA sheared to obtain an insert size of 350 bp. Libraries were then prepared using the Illumina genomic Nano kit. Sequencing was performed on the Illumina NovaSeq 6000 platform. Five libraries of the 117 DNA extracts needed to be repeated, resulting in a total of 122 metagenomes. All scripts and computer code used for the metagenomic data analysis are included in the release of the git repository on Zenodo^34^. In the following, if not stated otherwise, default settings were used. More details on parameter settings (including defaults) can be found in the scripts mentioned above.

**Fig. 3:**
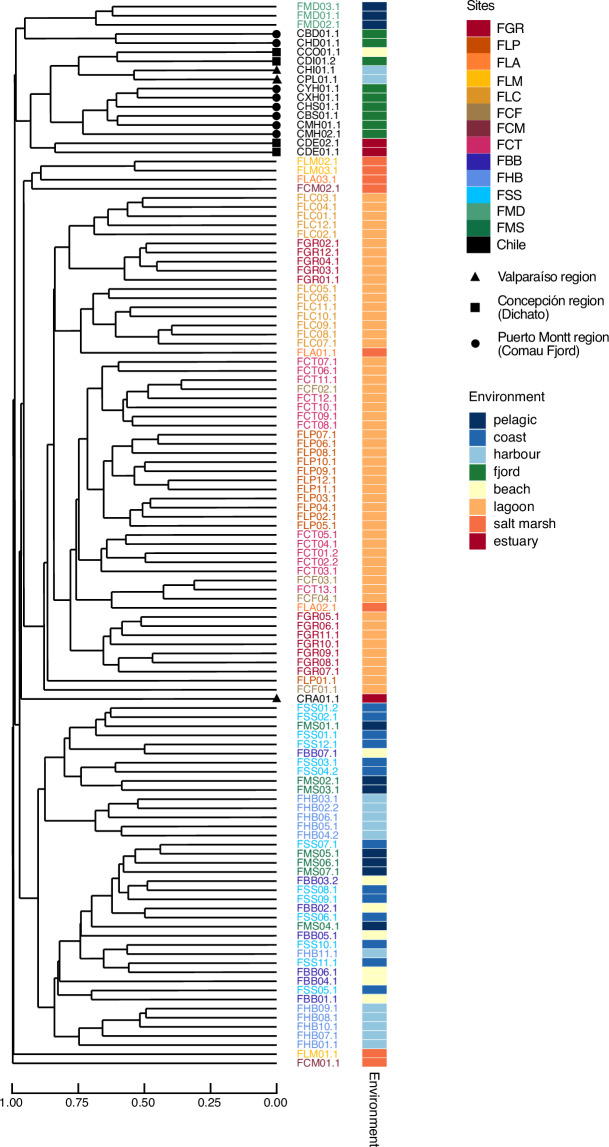
Hierarchical clustering of metagenomic samples (k-mer-based Bray-Curtis dissimilarity of clean reads and average linkage clustering). Label colors indicate the site samples derived from, marks at branch tips indicate the region Chilean samples derived from, and the colored strip indicates the environment samples derived from.

Metagenomic read quality control was conducted with bbduk, implemented in bbmap v39.10^44^. PhiX and adapter sequences were removed with a kmer length of 28 and 23 bp (mink = 11), respectively. Optical duplicates were filtered out with 2 allowed substitutions using distance settings specific for NovaSeq reads. Then, quality trimming was performed with a minimum average Phred score cut-off of 15 within a 4 bp sliding window, a minimum read length of 100 bp and a maximum poly-G tail length of 10 bp. Read quality was confirmed using fastqc v0.12.1 (https://github.com/s-andrews/FastQC). The data from the resequenced samples (ERR15116679 = ERR15116882, ERR15116680 = ERR15116681, ERR15116723 = ERR15116724, ERR15116672 = ERR15116673, ERR15116691 = ERR15116692) was then combined after quality trimming. Human reads were identified using bmtagger v3.101^45^ and removed using the script skip_human_reads.py of metaWRAP^46^ resulting in the clean reads used for all further processing steps. Metagenomic coverage was estimated with nonpareil v3.5.5^47^ using the kmer algorithm. Beta diversity (Bray-Curtis dissimilarity) was calculated based on kmer frequencies using SimkaMin v1.5.3^48^ with a kmer size of 31 bp to establish an assembly strategy based on average linkage hierarchical clustering in R v4.4.2^40^ using the vegan package v.2.6-10^49^. As there was no consistent clustering according to geographic location or habitat type (Fig. 3), a single *de novo* co-assembly was computed from all metagenomic samples using megahit v1.2.9^50^ with a minimum contig length of 1000 bp and a minimum multiplicity for filtering of 2, iterating over kmers 29, 39, 49, 59, 69, 79, 89, 99, 109, 119, 129, and 141. The co-assembly strategy was chosen to improve recovery of low-abundant genomes and increase the assembly’s completeness and contiguity. Additionally, *de novo* assemblies were computed with spades v4.0.0^51^ at kmers 21, 33, 55, 77, 99 and 127 for each single sample to best capture strain-level variations. For further processing, contigs <1,000 bp (co-assembly) and <500 bp (single-sample assembly) were removed and fasta headers were simplified using anvi’o v.8^52^.

Clean reads of each sample were mapped onto each of the assemblies with bowtie2 v2.5.4^53^ and filtered using samtools v1.21^54^, removing supplementary alignments and those shorter than 50 bp. The co-assembly and single-sample assemblies were binned using metabat2 v2.17^55^ with a minimum contig length of 1,500 bp and concoct v1.1.0^56^ with a minimum contig length of 1000 bp. Single-sample assemblies were additionally binned using vamb v5.0.4^57^, and semibin2 v2.2.0^58^ with a minimum contig length of 1,000 bp. A refined and consolidated set of prokaryotic bins for each assembly was obtained with the metaWRAP^46^ bin refinement module at a minimum completeness and maximum contamination threshold of 50% and 10%, respectively, according to checkM v1.2.2^59^. After bin refinement, bins from all assemblies were combined into one non-redundant bin set by dereplication using dRep v3.5.0^60^ with the ANImf algorithm, a primary average nucleotide identity (ANI) threshold of 0.9 (mash^61^) and a secondary ANI threshold of 0.98 (fastANI^62^) with a minimum aligned fraction of 0.5. Dereplication was validated using fastANI v1.34 with a fragment length of 1000 bp and differential coverage information computed with coverm v0.7.0^63^ using a minimum read percentage identity of 95% and minimum read aligned length of 50 bp to merge previously undetected redundant clusters using a custom script. To improve bin quality, each non-redundant bin was reassembled using a modified version of the metaWRAP reassembly module that takes both the clean reads recruited by the respective cluster representative bin and the clean reads recruited by all redundant members of the bin cluster for separate reassemblies, allowing for either 2 (strict) or 5 (permissive) mismatches in the read alignment. Of all five versions of each non-redundant bin (original, strict representative bin, permissive representative bin, strict all cluster members, permissive all cluster members), the best bin assembly was selected based on checkM completeness and contamination estimates. Furthermore, all bins with a completeness of at least 70% and a N50 value of >10,000 bp were additionally manually refined using anvi’o v8^52^. The quality of obtained MAGs was calculated with checkM2 v1.1.0^64^. Taxonomic assignment was performed with gtdbtk v2.4.1^65^ using the GTDB database version R226^42^. Gene prediction and functional annotation was performed with bakta v1.11.2^66^ in metagenome mode to identify genetic novelty. Unknown proteins were defined as genes encoding hypothetical proteins without any Pfam^67^ or UniRef^68^ hits according to the bakta annotation.

In parallel to the binning, a gene catalogue was created to assess functional diversity independent of the genomic context. For this, contigs from all assemblies were sorted by taxonomic domain using a consensus classification based on TIARA v1.0.3^69^ and DeepMicroClass v1.0.5^70^. Briefly, the classification based on DeepMicroClass was used for contigs of unknown origin according to the first stage TIARA prediction performed at a probability cut-off of 0.6. Furthermore, DeepMicroClass classifications were preferred over those of TIARA for viruses and contigs shorter than 3000 bp. Finally, contigs classified as organelle, prokaryote and prokaryotic virus were grouped as contigs presumably of prokaryotic origin, while those classified as eukaryote and eukaryotic virus were grouped as contigs presumably of eukaryotic origin. Genes were then predicted with prodigal v2.6.3^71^ for prokaryotic contigs and metaEuk v7.bba0d80^72^ for eukaryotic contigs. For each taxonomic domain, a non-redundant gene set was created using mmseq2 v17.b804f^73^ at 95% amino acid similarity and 90% coverage of the shorter sequence using greedy incremental clustering. Non-redundant prokaryotic and eukaryotic genes were quantified separately with salmon v1.10.3^74^ using the coding sequences of the respective other taxonomic domain as decoys in the index. Gene abundances are reported as gene length-normalized fragment counts per million analogous to TPM (transcripts per million). To provide an overview of the functional potential as well as functional novelty of the microbial communities, the gene catalogue was annotated with KOfam-scan v1.3.0 using the KEGG release 114^75^ and with diamond blastp v2.16.0 against Uniref100 released on 18.06.2025^68^. Gene novelty was defined when no hit to either of the two databases was detected.

## Data Record

Sequence data for this study and the anvi’o refined MAG set have been deposited in the European Nucleotide Archive (ENA) at EMBL-EBI under study accession PRJEB90443^76^, using the data brokerage service of the German Federation for Biological Data (GFBio^77^), in compliance with the Minimal Information about any (X) Sequence (MIxS) standard^78^. In addition, all ASV data tables and MAGs retrieved in this study and the gene catalogue can be found on Zenodo^34^. Environmental data has been deposited in Pangaea^33^, brokered by GFBio^77^. Additional data tables used as input for figure generation, technical validations and environmental data tables are available on Zenodo^34^. A detailed description of its contents is included in this data record as well as in the README of the git repository documenting all analysis steps and output files (https://git.iow.de/bio_inf/IOWseq000048_gesifus.field).

## Technical Validation

### Metabarcoding dataset

**Fig. 4:**
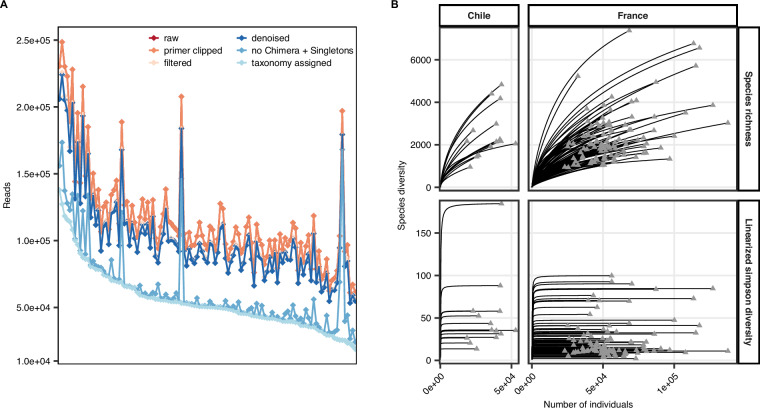
Quality statistics of the metabarcoding data. (**A**) Number of reads after individual processing steps of the dada2 pipeline. Samples were sorted based on the final number of sequences after taxonomic assignment. (**B**) Rarefaction curves for species richness and inverse Simpson index of individual samples from Chile and France.

Metabarcoding was conducted to obtain first insights into the taxonomic composition of the microbial community at the sampled sites. After the bioinformatic processing of the raw reads, between 1.9 × 10^4^ and 1.4 × 10^5^ sequences per sample were retained corresponding to 13–73% of the raw reads (Fig. 4A). Notably, samples FLM02, FLM03, and CPL01 contained high proportions of sequences assigned to chloroplasts (84.8%, 63.6%, and 62.8%), which resulted in the low number of final sequences compared to raw reads. Rarefaction analysis revealed that species richness estimates continued to increase with sequencing depth, suggesting the presence of undetected rare taxa (Fig. 4B). However, the asymptotic behaviour of the inverse Simpson index across all samples indicated that the diversity of the dominant members of the community was well covered (Fig. 4B). Thus, sequencing effort was sufficient to capture major ecological signals in the community.

### Metagenomic dataset

**Fig. 5:**
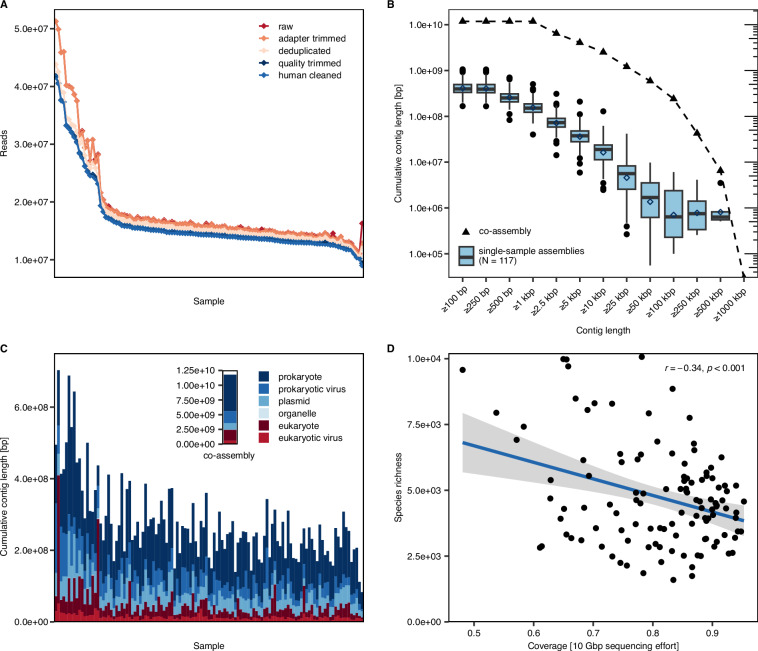
Quality statistics of metagenomic data. (**A**) Number of reads after individual processing steps of the metagenomic pipeline. Samples were sorted based on the final number of clean reads that were further used for computing *de novo* assemblies. (**B**) Cumulative contig lengths of metagenomic assemblies contained in contigs of increasing minimum size. Boxplots show the interquartile range with median (black bar) and mean (empty circles), and whiskers representing minimum and maximum without outliers (filled circles) defined at 1.5 times the interquartile range removed from the lower and upper quartiles, respectively. (**C**) Cumulative contig lengths by predicted taxonomic domain in single-sample assemblies and the co-assembly. Samples displayed in the same order as in plot (A). (**D**) Species richness of samples computed from metabarcoding data compared to metagenomic coverage at 10 Gbp sequencing effort. Pearson correlation statistics are displayed in the figure.

Metagenomic sequencing was performed in addition to metabarcoding to complement community-based information with gene- and genome-level functional information. Overall, the number of clean reads per sample ranged from 9 × 10^6^ to 4.2 ×10^7^ read pairs, corresponding to a loss of 8–45% compared to the raw data (Fig. 5A). Structural consistency between the metabarcoding and metagenomic datasets was assessed using a Mantel test^79^ on Bray-Curtis dissimilarity matrices based on ASV proportions and k-mer frequencies of clean reads, respectively. The test, implemented in the R package vegan (v.2.6–10 in R v4.2.2), yielded a strong correlation (Pearson r = 0.93, p = 0.001).

The cumulative length of contigs in computed *de novo* single-sample assemblies after removal of contigs <500 bp was 83–703 Mbp (Fig. 5B). In single-sample assemblies, up to 29 contigs with a contig length >100 kbp (FHB03) and up to 4 contigs with a contig length >500 kbp were detected (FLC03). The cumulative contig length of contigs >1 kbp for the co-assembly reached 11.8 Gbp. A total of 1,532 contigs were >100 kbp with a cumulative length of 240 Mbp, including 10 contigs >500 kbp with a cumulative length of 6.5 Mbp.

Overall, 33 to 65% of the cumulative contig length in single-sample assemblies was predicted to be of prokaryotic origin. The cumulative contig length assigned to prokaryotic viruses was especially high in assemblies from samples of sites FCF (28–39%), FCT (14–36%), and FLP (16–36%) (Fig. 5C, Table S2). Notably, more than 25% of the cumulative contig length was predicted to be of eukaryotic origin in assemblies of sites CDI01, CRA01, FCT06, FCT10, FLM01, FLM02 and FLM03, with highest values in assemblies from sites FLM02 (58.6%) and FLM03 (50.9%), consistent with the high proportion of chloroplast 16S rRNA gene amplicons detected in these samples as indicator for the presence of eukaryotic phytoplankton.

**Fig. 6:**
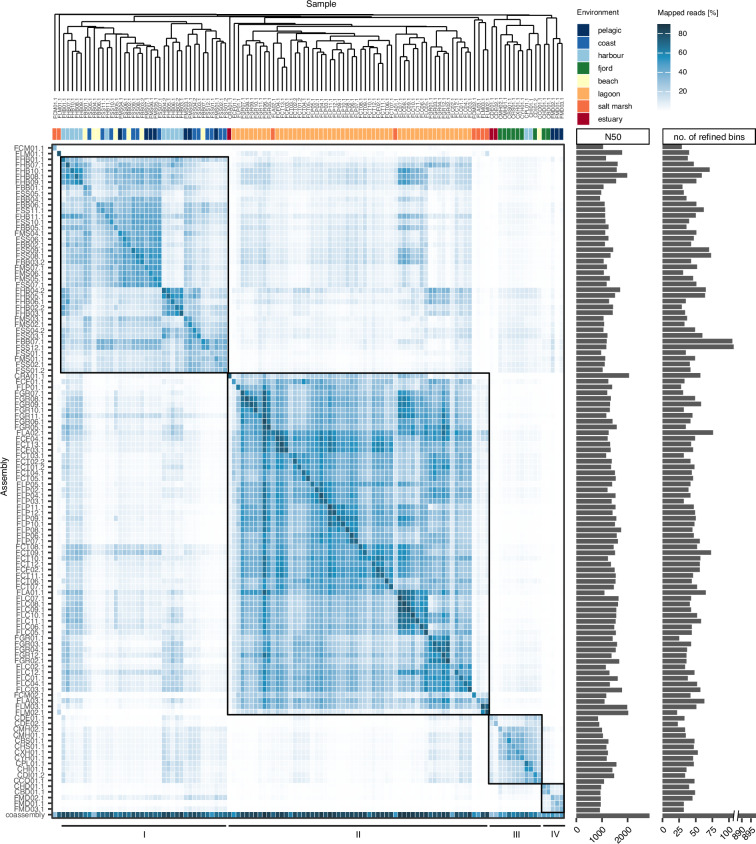
Mapping statistics for all metagenomic samples. Heatmap of mapped reads indicating the percentage of reads from each single sample (columns) mapped onto each assembly (rows). Metagenomic samples were ordered based on hierarchical clustering (k-mer-based Bray-Curtis dissimilarity of clean reads and average linkage clustering). A more detailed dendrogram is provided in Fig. 3. Coloured strips indicate the sampled environments. The four major clusters are indicated as black rectangles. N50 statistics of each assembly are indicated. Additionally, the number of metaWRAP-refined prokaryotic bins is displayed for each assembly.

Moreover, we demonstrate a correlation between species richness based on metabarcoding and the estimated coverage of metagenomes at 10 Gbp sequencing effort, despite substantial noise in the data (Fig. 5D). Still, while the metagenomic coverage of some samples with higher species richness was comparably low, the majority of samples achieved a coverage of >60% at the available sequencing depth. This high metagenomic coverage is reflected in the good quality of metagenomic assemblies as assessed by N50 and read recruitment (Fig. 6).

To gain insights into read recruitment of all samples and evaluate potential assembly strategies for future projects, we performed cross-mapping of all samples onto all single-sample assemblies and the co-assembly (see also Usage Notes). The hierarchical clustering of metagenomic samples in combination with the proportion of reads recruited by the single-sample assemblies revealed three major clusters: (I) samples from sites of France that were retrieved from pelagic or coastal environments, harbours and beaches, (II) samples from lagoon sites in France and one site in Chile from an estuary system (CRA01), (III) samples from sites in Chile that were retrieved from estuaries, fjords, harbours and the beach. In addition, samples from France and Chile (CBD01, CHD01, FMD01-03) that were retrieved from deep-sea water samples clustered together in a fourth smaller cluster (IV) (Figs. 3, 6). Two samples from France derived from salt marshes (FCM01 and FLM01) did not cluster with any of the other lagoon samples. Within individual clusters, the proportion of mapped reads ranged from 0.35% to 82%. The high proportions were obtained from the mapping of the original reads used for the *de novo* assembly back onto the respective single-sample assemblies and thus reflect a high assembly quality. The N50 values of single-sample assemblies with a minimum contig length of 500 bp reached up to 2,057 bp, with samples from deeper water masses exhibiting lower N50 values, ranging from 846 to 968 bp. The co-assembly achieved a N50 of 2,856 bp but at a minimum contig length of 1,000 bp.

### Final set of bins

**Fig. 7:**
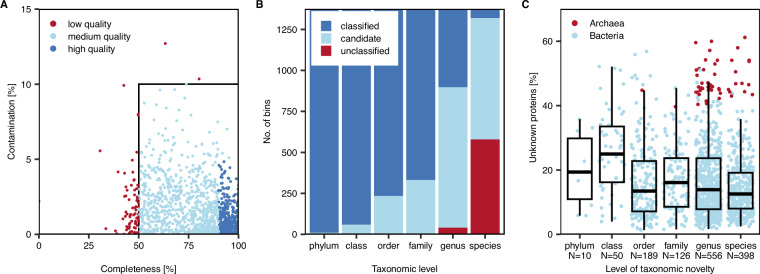
Final bin statistics. (**A**) Completeness and contamination of individual bins excluded from the final MAG set due to low quality based on checkM2, and of bins included in the final MAG set, comprising medium-quality MAGs, and high-quality MAGs. (**B**) Number of classified, candidate and unclassified MAGs per taxonomic level. (**C**) Proportion of unknown genes per MAG sorted by the level of taxonomic novelty. MAGs of the domains Archaea and Bacteria are indicated by color.

The number of metaWRAP-refined bins ranged from 22 to 107 for single-sample assemblies and reached 898 bins for the co-assembly. Overall, the mean completeness of refined bin sets ranged from 64 to 81%. The non-redundant bin set after bin dereplication contained 1,448 bins with a mean completeness of 77.5% and mean contamination of 2.7%. After reassembly, 1,144 bins improved in completeness and contamination, increasing mean bin completeness by 2.7 points and decreasing mean contamination by 1 point. Notably, the completeness and contamination results between the checkM and checkM2 outputs for the reassembled bin set partially differed by up to 53 points in completeness, and 76 (5%) of the bins from the reassembled bin set did not meet the quality criteria for medium quality MAGs according to MIMAG^23^ (>50% completeness, <10% contamination) based on checkM2 (Fig. 7A, Table S5). From this final MAG set (1,372 bins), we manually curated a total of 763 MAGs in anvi’o, of which 468 were of high quality based on checkM2 (completeness >90%, contamination <5%) (Table S6).

Prokaryotic taxonomic novelty within the final MAG set was determined by the absence of assignment to a classified taxon at a given taxonomic rank. While at the phylum level, 99.3% of all MAGs were assigned to classified taxa, decreasing to 35% at the genus level (Fig. 7B), at the species level, only 4% of all MAGs were assigned to classified taxa. The remaining MAGs affiliated with either unclassified (42%) or candidate taxa (54%).

To evaluate genetic novelty in individual MAGs, the proportions of unknown proteins (definition see Methods: Metagenomic sequence processing) were calculated. We observed that on average the proportion of unknown proteins was higher in MAGs of lower taxonomic resolution (candidate phyla, candidate classes) compared to those of higher taxonomic resolution. Besides this general observation, genetic novelty was most prominent in MAGs affiliated with the bacterial phylum Verrucomicrobiota as well as archaea of the phyla Thermoplasmatota and Thermoproteota, reaching up to 61% of unknown proteins within individual MAGs, even if classified at genus or species level (Fig. 7C, Table S7). Patescibacteriota (up to 57%), Myxococcota (up to 51%), members of the Pseudomonatota (Alphaproteobacteria, Gammaproteobacteria, up to 51%), Bacteroidota (up to 47%) and Babelota (up to 45%) also exhibited high proportions of unknown proteins.

### Gene catalogue

**Fig. 8:**
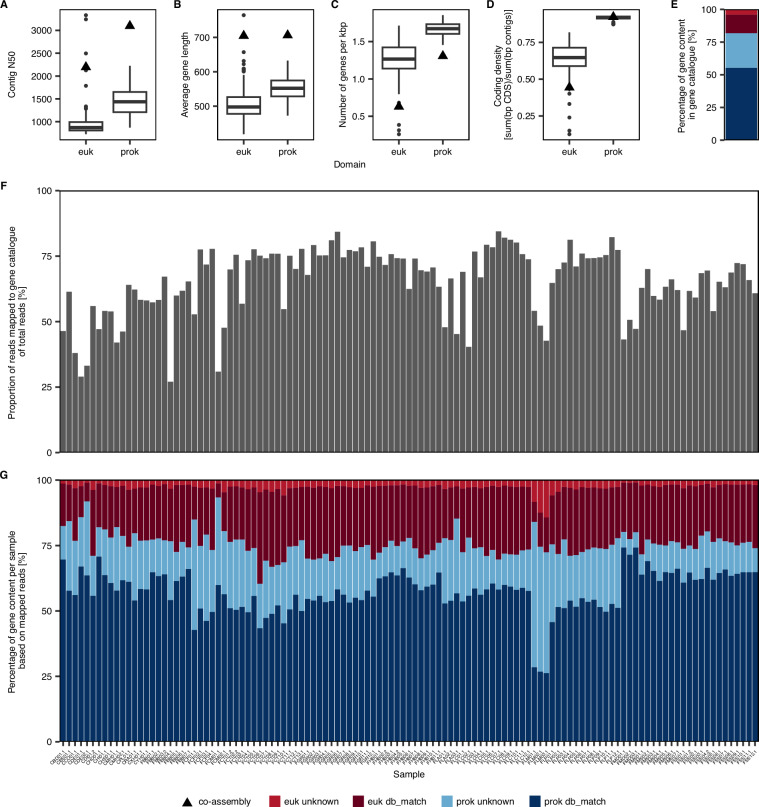
Gene catalogue statistics. (**A**) N50 of prokaryotic (prok) and eukaryotic (euk) contigs. (**B**) Average gene length. (**C**) Number of genes per kbp contigs. (**D**) Coding density. (**E**) Proportion of prokaryotic and eukaryotic annotated (db_match) and unannotated (unknown) proteins in the non-redundant gene catalogue. (**F**) Proportion of clean reads mapped to the gene catalogue. (**F**) Proportion of prokaryotic and eukaryotic annotated (db_match) and unannotated (unknown) proteins per sample.

We predicted between 126,152 and 934,305 genes on prokaryotic contigs and between 15,114 and 167,794 genes on eukaryotic contigs per single-sample assembly, with 12,236,660 and 1,533,527 genes predicted on the co-assembly, respectively. This corresponded to a frequency of approximately 1.6 prokaryotic and 1.2 eukaryotic genes per kbp of assembled contigs. Furthermore, eukaryotic genes were on average shorter than prokaryotic ones resulting in a lower gene density of 0.65 compared to 0.92 (Fig. 8). However, the observed differences between prokaryotic and eukaryotic gene statistics may also have been exaggerated by a technical bias affecting mainly the eukaryotic gene prediction, thereby leading to an underestimation of gene length, frequency, and density. Eukaryotic contigs exhibited a lower N50 than prokaryotic ones, indicative of a more fragmented assembly, which may have resulted in a larger fraction of incomplete or unidentified genes. Additionally, unlike prokaryotic genes which were predicted *de novo*, eukaryotic genes were identified using a reference-based strategy. This approach may underperform in understudied environments with high taxonomic and functional novelty as observed in the present dataset.

After clustering at 95% amino acid similarity, we obtained a non-redundant gene catalogue consisting of 18,861,522 prokaryotic (82%) and 4,268,297 eukaryotic genes (18%). Overall, 31% of all non-redundant genes had no match to KEGG orthologs or Uniref100. Notably, the percentage of such unknown genes was lower for eukaryotic (27%) than for prokaryotic genes (49%), presumably related again to the dependency on a reference database for gene prediction. We assume that the fraction of functional novelty may be considerably higher for eukaryotes than we can estimate with currently available methods.

In terms of read counts, 58–93% of the mapped reads per sample were assigned to prokaryotic genes with 5–55% being recruited by those without functional annotation. Meanwhile, 7–42% of mapped reads were assigned to eukaryotic genes with 1–15% mapping to those of unknown function. Samples from the FLM station, which were previously highlighted due to their high proportion of chloroplast amplicon sequences as well as their large contribution of eukaryotic contigs to the assembly, exhibited by far the highest percentage of both eukaryotic and prokaryotic unknown proteins. The high proportion of unknown proteins at this site may be attributable to the elevated salinity levels and fluctuations, which ranged from 46 to 118 practical salinity units (PSU) in three samples collected within three months, characterizing this station as a rarely studied extreme environment. To provide some perspective on the read count statistics, we further expressed read recruitment to the non-redundant gene catalogue as a fraction of the total number of clean reads per sample. While for the majority of samples (n = 99) more than 45% of the clean reads were recruited by the gene catalogue, the mapping rate ranged from 24% to 44% for a substantial number of samples (n = 18).

## Usage Notes

The metagenomic dataset consists of a total of 117 sequenced DNA extracts from 116 samples, of which five samples were resequenced (see Methods: Metagenomic sequence processing). Sample FSS01 was sequenced twice from two replicate DNA extracts, FSS01.1 (SAMEA118554837) and FSS01.2 (SAMEA118554836). As those two metagenomic samples clustered closely together (Fig. 6), for further usage, reads from these samples could be combined. In addition, mapping statistics revealed that instead of one large co-assembly using all samples, co-assemblies using one of the aforementioned clusters could be used (see also Technical Validation) to target specific environments represented in this dataset. It should be considered when comparing samples from France and Chile that these samples are derived from different sampling time points, potentially confounding space and time. Furthermore, we performed a test on the performance of metagenomic binners using this dataset. An overview of utilized binners, total numbers of bins per binner and medium quality bins (>50% completeness, <10% contamination) can be found in Table S4. Overall, CONCOCT performed poorly on the computed co-assembly with only 1% of bins being of at least medium quality, compared to 27% of bins using metabat2. These results are consistent with previous observations using CONCOCT on complex datasets^46^. Other tested binners, such as semibin2 or comebin did not finish within reasonable time for the co-assembly (run aborted after one week). Thus, we suggest to perform binning of larger co-assemblies of this dataset with metabat2 only. For single-sample assemblies, most bins of medium quality were obtained using semibin2.

## Supplementary information

Supplementary tables

## Data Availability

Sequence data for this study and the anvi’o refined MAG set have been deposited in the European Nucleotide Archive (ENA) at EMBL-EBI under study accession PRJEB90443^76^, using the data brokerage service of the German Federation for Biological Data (GFBio^77^), in compliance with the Minimal Information about any (X) Sequence (MIxS) standard^78^. In addition, all ASV data tables and MAGs retrieved in this study and the gene catalogue can be found on Zenodo^34^. Environmental data has been deposited on Pangaea^33^, brokered by GFBio. Additional data tables used as input for figure generation, technical validations and environmental data tables are available on Zenodo^34^.

## Code availability

All code used to perform bioinformatic analyses, technical validations and data preparations can be found on https://git.iow.de/bio_inf/IOWseq000048_gesifus.field with the release associated with the state of the manuscript at the time of acceptance for publication available on Zenodo^34^.
